# Quantifying “Medical Renal Disease”: A Pediatric Pilot Study Using Ultrasound Radiomics for Differentiating Acute Kidney Injury and Chronic Kidney Disease

**DOI:** 10.3390/diagnostics15162112

**Published:** 2025-08-21

**Authors:** Laura De Leon-Benedetti, Laith R. Sultan, Hansel J. Otero, Tatiana Morales-Tisnés, Joya Sims, Kate Fitzpatrick, Julie C. Fitzgerald, Susan Furth, Benjamin L. Laskin, Bernarda Viteri

**Affiliations:** 1 Radiology Department, Children’s Hospital of Philadelphia, 3401 Civic Center Blvd., Philadelphia, PA 19146, USA; sultanl@chop.edu (L.R.S.); oteroh@chop.edu (H.J.O.); moralest@chop.edu (T.M.-T.); viterib@chop.edu (B.V.); 2 Perlman School of Medicine, University of Pennsylvania, Philadelphia, PA 19104, USA; joya.sims@pennmedicine.upenn.edu (J.S.); fitzgeraldj@chop.edu (J.C.F.); furths@chop.edu (S.F.); laskinb@chop.edu (B.L.L.); 3 Sidney Kimmel Medical College, Thomas Jefferson University, Philadelphia, PA 19107, USA; kate.fitzpatrick@students.jefferson.edu; 4 The Pediatric Intensive Care, Department of Anesthesiology and Critical Care Medicine, Children’s Hospital of Philadelphia, Philadelphia, PA 19104, USA; 5 Research Institute, Children’s Hospital of Philadelphia, Philadelphia, PA 19146, USA; 6 Division of Nephrology, Children’s Hospital of Philadelphia, Philadelphia, PA 19104, USA

**Keywords:** nephrology, diagnostic ultrasound, radiomics, acute kidney injury, chronic kidney disease

## Abstract

**Background:** Differentiating acute kidney injury (AKI) from chronic kidney disease (CKD) in children remains a critical unmet need due to the limitations of current clinical and biochemical markers. Conventional ultrasound lacks the sensitivity to discern subtle parenchymal alterations. This study explores the application of ultrasound radiomics—a novel, non-invasive, and quantitative image analysis method—for distinguishing AKI from CKD in pediatric patients. **Methods:** In this retrospective cross-sectional pilot study, kidney ultrasound images were obtained from 31 pediatric subjects: 8 with oliguric AKI, 14 with CKD, and 9 healthy controls. Renal parenchyma was manually segmented, and 124 advanced texture features were extracted using the open-source ©PyFeats. Features encompassed multiple categories (e.g., GLCM, GLSZM, WP). Statistical comparisons evaluated intergroup differences. Principal Component Analysis identified the top 10 most informative features, which were used to train supervised machine learning models. Model performance used five-fold cross-validation. **Results:** Radiomic analysis revealed significant intergroup differences (*p* < 0.05). CKD cases exhibited increased echogenicity and heterogeneity, particularly in GLCM and GLSZM features, consistent with chronic fibrosis. AKI cases displayed more homogeneous texture, likely reflecting edema or acute inflammation. While echogenicity separated diseased from healthy kidneys, it lacked specificity between AKI and CKD. Among ML models, XGBoost achieved the highest macro-averaged F1 score (0.90), followed closely by SVM and Random Forest, demonstrating strong classification performance. **Conclusions:** Radiomics-based texture analysis of grayscale ultrasound images effectively differentiated AKI from CKD in this pilot study, offering a promising, non-invasive imaging biomarker for pediatric kidney disease. These preliminary findings justify prospective validation in larger, multicenter cohorts.

## 1. Introduction

The high incidence of acute kidney injury (AKI) among hospitalized children is associated with the need for mechanical ventilation, prolonged hospital stays, higher mortality, and substantial morbidity [1]. Among children with AKI in the intensive care unit, 6% require kidney replacement therapy [2]. Despite this, it is often under-recognized [3]. Current diagnostic markers for AKI are notably suboptimal and make the distinction between acute, chronic, and acute-on-chronic conditions in patients with new-onset kidney disease even more challenging. Biological markers can guide this distinction, but are not always accurate, and the differential diagnoses can still be broad and require a blood draw (invasive). Prompt identification of AKI can activate appropriate interventions to minimize tissue damage. There is a need for a reliable, non-invasive diagnostic tool that can quantify damage, accurately identify AKI, and distinguish AKI-induced parenchymal alterations from those observed in chronic kidney disease (CKD).

Differentiating AKI from CKD in children with new-onset kidney dysfunction presents a significant diagnostic challenge. Common biochemical markers—such as serum creatinine and cystatin C—are limited by delayed response and susceptibility to non-renal influences like muscle mass and systemic inflammation [4,5,6,7,8]. Although emerging biomarkers such as neutrophil gelatinase-associated lipocalin (NGAL) offer promise for early detection of severe AKI, they remain underutilized and lack specificity in distinguishing AKI from CKD [5,7,9,10]. Even tools like intact parathyroid hormone measurements, while occasionally helpful, suffer from limited precision and still require invasive sampling. As a result, clinicians often face a broad differential without clear, non-invasive diagnostic tools to guide early clinical decisions.

While MRI-based techniques have been explored to evaluate kidney fibrosis, their high cost, limited accessibility, and the need for sedation in young or critically ill children reduce their utility in routine pediatric care. Ultrasound, in contrast, is widely available, non-invasive, and remains the first-line imaging modality in pediatric nephrology due to its safety and affordability [11,12,13]. It is routinely used to assess kidney size, parenchymal echogenicity, and corticomedullary differentiation and to exclude obstructive causes of AKI. However, conventional sonographic features often lack the sensitivity and specificity required to distinguish AKI from CKD. Moreover, qualitative interpretations introduce variability and reduce diagnostic reliability, especially when evaluating disease severity or reversibility—challenges that are particularly important in the pediatric population, where minimizing invasiveness is critical [13,14]. 

Ultrasound radiomics—a post-processing, quantitative analysis approach—offers a promising solution by extracting high-dimensional data from conventional grayscale images by evaluating spatial intensity features such as tissue heterogeneity, organization, and density [15]. Algorithms such as the gray-level co-occurrence matrix (GLCM) and run-length matrix (RLM) provide insight into the spatial distribution and frequency of pixel intensities, capturing microstructural alterations in the tissue [9,16]. These advanced texture features may improve diagnostic precision and overcome the limitations of conventional ultrasound in differentiating AKI from CKD. We propose a novel, computer-aided method for kidney texture analysis based on spatial intensity patterns observed in ultrasound images. This radiomics-based approach aims to provide a non-invasive, reliable, and scalable diagnostic tool for identifying AKI and distinguishing it from CKD. In this study, we further evaluate the potential of radiomics in differentiating AKI, CKD, and healthy controls.

## 2. Materials and Methods

### 2.1. Study Design and Selection Criteria

This is an IRB-approved retrospective study involving kidney ultrasound images from three different groups: oliguric AKI (Kidney Disease: Improving Global Outcomes [KDIGO] stage 3 and not on dialysis), CKD, and healthy controls (Table 1) [1]. The mean estimated glomerular filtration rate (eGFR) for each group was measured using the U25 CKiD formula in mL/min/1.76m^3^ and was representative of the subjects’ kidney dysfunction [4]. The sample size was determined based on practical considerations and prior recommendations for pilot studies.

Acute kidney injury cases were retrospectively identified and confirmed through medical record review. Indications for imaging were oliguric/anuric AKI with elevated serum creatinine. Acute kidney injury patients who required dialysis had imaging prior to dialysis initiation. Patients had a variety of primary medical conditions, not all primary kidney in nature, such as liver transplant failure, post-cardiac surgery, severe hypernatremia, possible cortical necrosis in the setting of cardiac arrest, renal tubular dysgenesis (ACE gene mutation), and vasculitis. Chronic kidney disease cases and healthy controls were identified from the NIH-approved prospective cohort studies: the Chronic Kidney Disease in Children study (CKiD) [5] and the Imaging Modalities in Pediatric Assessment of Kidney Transplants (IMPAKT) study [6], respectively. Healthy controls of the IMPAKT study are identified at random through a volunteer database of the recruitment enhancement core at the Children’s Hospital of Philadelphia (see Table 1 for selection criteria).

### 2.2. Quantitative Ultrasound Image Analysis

Sixty-two two-dimensional (2D) kidney B-mode ultrasound (US) images were selected retrospectively from the patient database. Ultrasound images were obtained with linear or curved probes commonly used for abdominal imaging by a clinical scanner. The selection of images for analysis was guided by factors that included US image quality, clarity of image, lack of annotations and measurements, and the best visualization of the kidney parenchyma, collecting system, and renal pelvis.

#### 2.2.1. Region of Interest Selection

**Figure 1:**
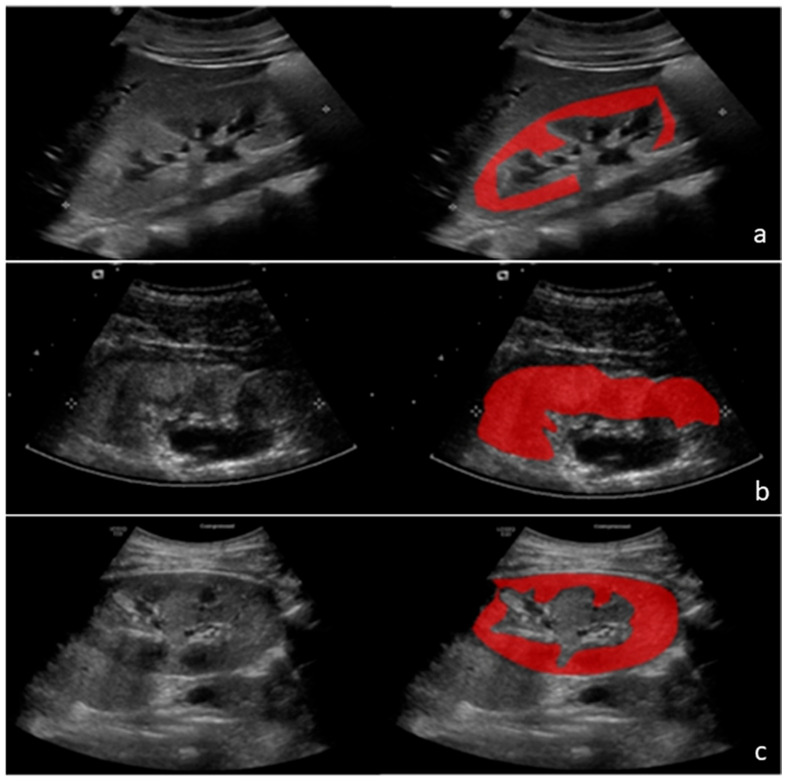
Illustrated examples of the three groups ((**a**) Healthy controls, (**b**) AKI, and (**c**) CKD). The ultrasound image of the kidney is shown side by side, followed by the same ultrasound image with the specified region of interest that we identified in red.

Regions of interest (ROIs) were manually delineated on B-mode ultrasound images (as depicted in Figure 1) and defined as the visible renal parenchyma, including both cortical and medullary regions, while excluding the renal pelvis and collecting system. ROIs were carefully traced along the outer boundary of the kidney, closely adhering to anatomical contours to ensure consistent coverage of the renal parenchyma. Special attention was paid to excluding areas affected by common ultrasound imaging artifacts, such as acoustic shadowing, posterior enhancement, reverberation artifacts, and regions with poor visibility. Segmentation was consistently performed on sagittal long-axis views, providing optimal visualization of the entire kidney and standardized anatomical orientation across all subjects. Manual delineation was performed by four trained research analysts (J.S., K.F., L.R.S., and L.D.L.-B.) and reviewed by a board-certified nephrologist and nephrology imaging specialist (B.V.) to verify anatomical accuracy and consistency. In instances where ROI boundaries were unclear or ambiguous, consensus was reached through collaborative discussion.

Before feature extraction, gray-level normalization was applied within each ROI by rescaling pixel intensities to a standardized range of 0–255 [7,8]. This min–max normalization approach ensured consistency in intensity distributions across different cases, thereby improving the comparability of texture features despite variability in ultrasound acquisition parameters.

#### 2.2.2. Feature Extraction

**Figure 2:**
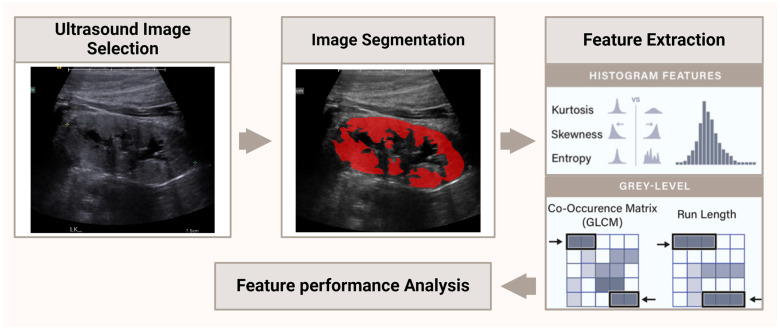
A schematic showing the process followed for image analysis, including image selection, image segmentation, and feature extraction, which lead to performance analysis.

A comprehensive radiomic feature extraction pipeline was implemented (see Figure 2), using *PyFeats* v1.0.11, an open-source Python package for image feature extraction developed by Giakoumoglou et al. [17]. A total of 124 features were computed from each region of interest (ROI), encompassing a broad range of intensity-, texture-, and frequency-domain descriptors (Supplementary Table S1). Feature categories included first-order statistics, gray-level co-occurrence matrix (GLCM), gray-level difference statistics (GLDS), neighborhood gray-tone difference matrix (NGTDM), statistical feature matrix (SFM), Law’s texture energy (LTE), fractal dimension texture analysis (FDTA), gray-level run length matrix (GLRLM), Fourier power spectrum (FPS), morphological shape features, gray-level size zone matrix (GLSZM), higher-order spectra (HOS), local binary pattern (LBP), and wavelet packet decomposition. First-order features described pixel intensity distributions using metrics such as mean, variance, skewness, entropy, and percentile-based thresholds. GLCM-derived features captured second-order spatial dependencies between pixel pairs and included contrast, correlation, homogeneity, angular second moment, cluster tendency, and information measures of correlation. GLDS, NGTDM, and SFM features provided additional statistical descriptions of local gray-level variations and spatial structures. Texture heterogeneity was further quantified using LTE and GLRLM features, including run-length nonuniformity, high gray-level run emphasis, and short-run emphasis. Frequency-based features were derived using Fourier and wavelet transforms to capture periodicity and multiscale texture information. Shape descriptors, such as area, circularity, and convexity, were extracted from the ROI masks to characterize morphology. This multi-class feature framework was used to enable robust quantification of structural and textural patterns in ultrasound images and served as the foundation for downstream statistical and machine learning analyses.

### 2.3. Statistical Analysis

Descriptive statistics were used to summarize the data, with counts and frequencies reported for categorical variables and means, medians, standard deviations (SD), and interquartile ranges (IQR) provided for continuous variables.

Radiomic feature discrimination was performed at the kidney level, with each kidney unit treated as an independent observation. Because the majority of individuals contributed two kidneys, we used linear-mixed-effects models to account for this clustering by including a random intercept for subject ID. Group (i.e., healthy controls, AKI, or CKD) was modeled as a fixed effect, with healthy controls used as the reference category.

To assess overall differences across groups, we applied a joint Wald test. We conducted post hoc pairwise comparisons to identify which specific group pairs differed significantly. These comparisons were based on estimated marginal means (least-squares means) obtained via the margins command in Stata (StataCorp. 2025. Stata Statistical Software: Release 19. StataCorp LLC: College Station, TX, USA). Bonferroni correction was applied to adjust for multiple comparisons and control the familywise error rate. This approach allowed us to validly compare radiomic features between groups while accounting for the repeated-measures structure of the data. A *p*-value ≤ 0.05 was considered statistically significant. The Shapiro–Wilk test was used to assess the normality of data distribution.

### 2.4. Feature Selection and Classification Model

To reduce dimensionality and identify the most discriminative features, Principal Component Analysis (PCA) was performed using the scikit-learn package in Python (version 1.4.2) [18]. Feature loadings from the first principal components were examined, and the top 10 features with the highest absolute contributions were selected for downstream modeling. To evaluate the clustering capacity of the selected features, we applied Uniform Manifold Approximation and Projection (UMAP) for nonlinear dimensionality reduction using the umap-learn library [19]. A two-dimensional UMAP projection was generated to visualize the separation among diagnostic groups. All visualizations were created using matplotlib and seaborn.

The selected features were used to train and evaluate four supervised machine learning models: Random Forest, Support Vector Machine (SVM) with a radial basis function kernel, Logistic Regression, and XGBoost. Model development and validation were conducted in Python using the scikit-learn and xgboost libraries [18]. A 5-fold cross-validation strategy was employed to estimate model generalizability. Performance metrics included mean classification accuracy and macro-averaged F1 score.

## 3. Results

### 3.1. Patient Characteristics

A total of 31 subjects were included in this study: 8 were categorized as AKI, 14 as CKD, and 9 as healthy controls. Table 2 summarizes the demographic and clinical data. There was no age difference between the AKI and CKD subjects (median age 3.5 years [IQR: 0–11.5] and 3.5 years [IQR: 0–6.8], respectively), while the healthy controls were much older (median age: 15.5 years [IQR: 12.8–21]). All groups had the same number of female subjects (n = 6, each). Images of the left kidneys from two patients were excluded from the analysis due to suboptimal image quality, which limited accurate ROI delineation and feature extraction. In total, 60 kidney units were analyzed.

### 3.2. Ultrasound Radiomics Comparison Across Study Groups

**Figure 3:**
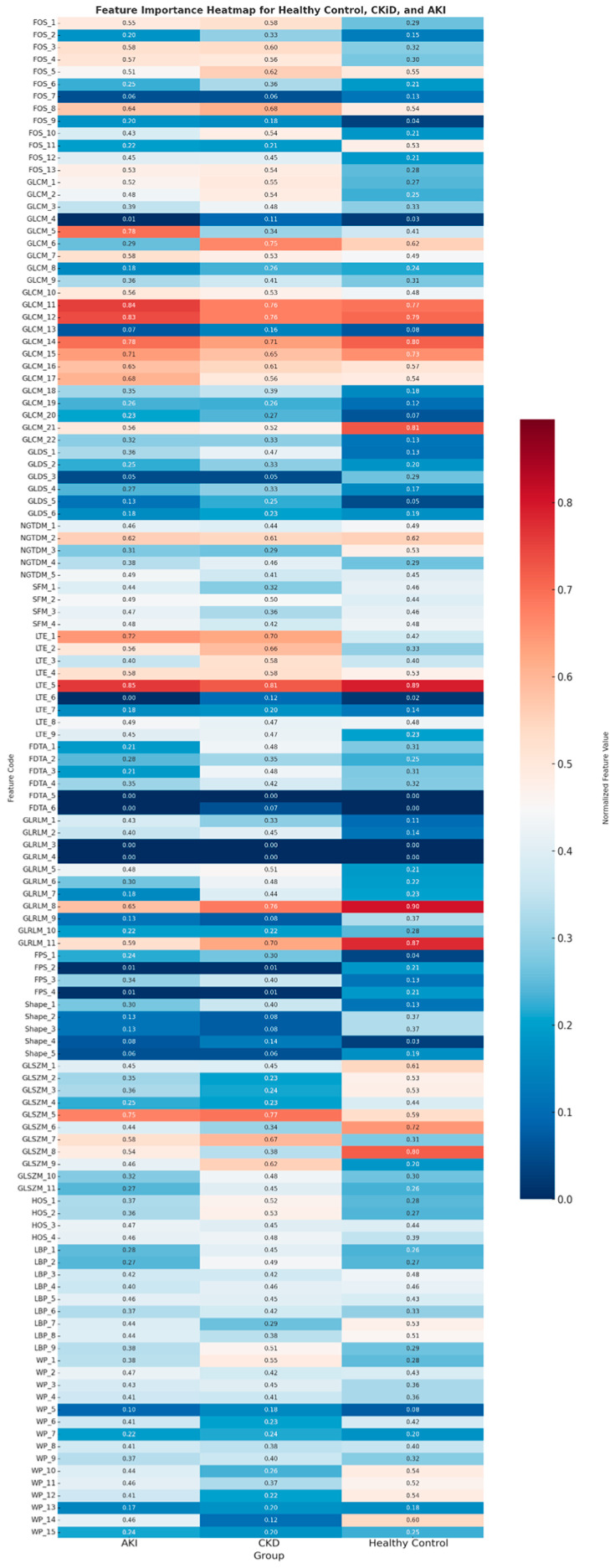
This heatmap illustrates the relative expression of the 124 radiomic features across the three study groups: AKI (Acute Kidney Injury), CKD (Chronic Kidney Disease), and Healthy Controls. Each row corresponds to a specific radiomic feature. The color scale represents the mean normalized value of each feature within each group, where lower values are shown in blue and higher values in red. Normalization was performed across the entire dataset to facilitate comparison on a common scale (e.g., z-score or min-max scaling). This visualization highlights patterns of feature expression and helps identify the most discriminative features across groups. A full feature code dictionary is available in Table S1.

Of the 124 features studied, 61 features showed significant differences between the three groups. Based on group comparisons, several radiomic features exhibited notable differences across the three groups: AKI, CKD, and healthy controls, which are illustrated in the heatmap in Figure 3.

Among the first-order histogram (FOS) features, echo intensity (FOS1) showed visibly higher values in AKI and CKD compared to the healthy control group. Although these differences were consistent among other FOS features, only a few FOS features showed substantial contrast between groups. For instance, Heterogeneity (FOS2) was higher in CKD compared to the other two groups. In the GLCM category, 8 out of 22 features (36.4%)—including GLCM_1, GLCM_2, GLCM_5, GLCM_6, GLCM_18, GLCM_20, GLCM_21, and GLCM_22—demonstrated significant intergroup differences. Diseased kidneys, particularly in the CKD group, exhibited consistently elevated GLCM values.

Run-length matrix (GLRLM) features were less consistent in group differentiation. Most features did not display significant differences; however, GLRLM_8 and GLRLM_11 stood out with higher normalized values in the healthy control group. For example, GLRLM_8 showed a value of 0.90 in controls, versus 0.65 in AKI and 0.76 in CKD. These findings suggest selective utility of specific run-length features (Figure 3).

The Gray Level Size Zone Matrix (GLSZM) features revealed substantial group-specific differences, with 10 out of 11 features (90.9%) demonstrating statistically significant intergroup variation. Notably, features such as GLSZM_5, GLSZM_6, and GLSZM_7 exhibited higher values in disease groups, particularly within CKD subjects. For instance, GLSZM_5 showed normalized values of 0.77 in CKD, compared to 0.75 in AKI and 0.59 in healthy controls (Figure 3).

In contrast, features from Wavelet Packet (WP), with only 3 out of 15 features (20%), and Local Binary Pattern (LBP), with 3 out of 9 features (33.3%), demonstrated more uniform distributions across the three diagnostic groups. These categories showed limited discriminatory value in distinguishing AKI, CKD, and healthy controls, as visually confirmed in the heatmap (Figure 3).

### 3.3. Radiomic Features Classification Model

Principal Component Analysis (PCA) identified the top 10 features with the highest absolute loadings on the first principal components, as seen in Table 3, which were then selected for downstream modeling. These features included a diverse representation from the GLCM, GLSZM, FDTA, GLDS, and Wavelet Packet (WP) categories, reflecting a combination of textural heterogeneity, spatial complexity, and frequency-domain characteristics (see Table 3).

**Figure 4:**
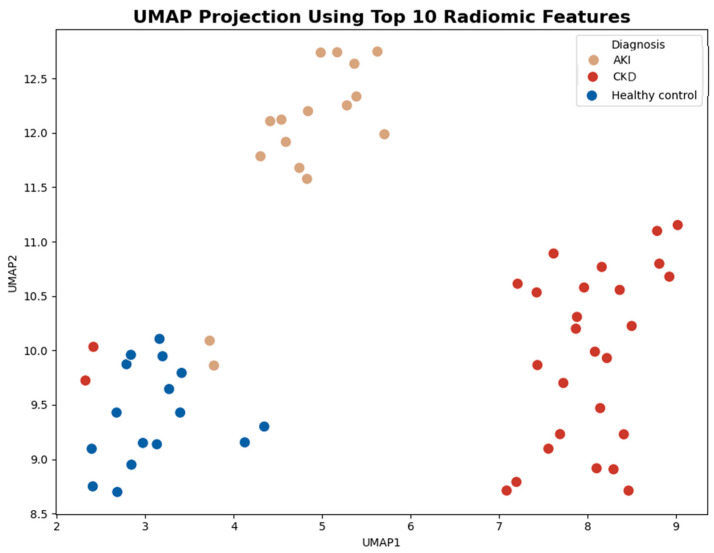
UMAP projection of kidney radiomic features across diagnostic groups.

The resulting two-dimensional UMAP embedding revealed clear and consistent separation among the three diagnostic groups—AKI, CKD, and healthy controls (Figure 4).

A two-dimensional UMAP (Uniform Manifold Approximation and Projection) plot visualizing the clustering of 60 kidney cases using the top 10 selected radiomic features. Each point represents a subject, colored by diagnosis: CKD (Chronic Kidney Disease)—dark red, AKI (Acute Kidney Injury)—beige, healthy controls—navy blue. The clear spatial separation of diagnostic groups indicates the discriminative power of the selected features in differentiating kidney pathology.

Using these features, all the supervised machine learning models achieved high performance in distinguishing between the diagnostic categories, as shown in Table 4. Among these, XGBoost demonstrated the highest macro-averaged F1 score (0.90), indicating robust classification performance across imbalanced classes.

## 4. Discussion

Accurate differentiation between kidney disease conditions is crucial. On the one hand, CKD is a progressive and often irreversible condition that requires long-term management focused on slowing disease progression and managing complications. In contrast, AKI is a potentially reversible condition that requires prompt intervention to restore kidney function and prevent long-term damage. The ability to accurately classify these conditions using non-invasive ultrasound imaging can help improve patient outcomes by facilitating earlier and more targeted interventions.

Texture features measure the spatial variation between the pixels, taking into account intensity and their relative position to one another, which could lead to a possible correlation between kidney texture and kidney injury [11,12]. As proposed by the liver studies, quantitative US texture features augment the diagnostic accuracy of B-mode ultrasound in identifying liver fibrosis, particularly in its early stages. This enhancement has proved crucial for distinguishing between initial and advanced stages of fibrosis and has successfully differentiated between normal, healthy tissue and diseased states [9].

The radiomic biomarkers identified in our study provide further microstructural insights and align with established pathophysiological changes in kidney disease. Our findings suggest that specific features are highly effective in distinguishing between CKD and AKI, providing valuable insights into the underlying differences in renal pathology. GLCM features capture the spatial relationships between pixel intensities, making them particularly sensitive to changes in tissue microarchitecture.

In CKD, characterized by fibrosis and glomerulosclerosis, our results revealed distinct radiomic signatures. Notably, CKD cases exhibited consistently higher values in features related to echogenicity and heterogeneity, particularly those derived from first-order statistics (e.g., FOS_5, FOS_6) and texture matrices such as GLCM and LTE. CKD showed higher importance in coarseness and zone-size metrics (e.g., GLSZM_1-3, NGTDM_2/3), consistent with chronic architectural remodeling and fibrosis. This increased heterogeneity reflects greater structural disruption where excessive collagen deposition leads to tissue irregularity [13,14]. These findings align with known sonographic characteristics of CKD, where increased cortical echogenicity and parenchymal heterogeneity are reflective of a more structurally disrupted tissue environment due to chronic fibrosis, scarring, and nephron loss.

In contrast, AKI, often associated with fluid accumulation and edema, tends to result in more homogeneous tissue [15,20]. The lower heterogeneity values in this stage, compared to CKD, indicate a more uniform tissue structure, linked to the acute inflammatory response. The AKI group demonstrated a clear pattern of elevated entropy-related features, including GLCM entropy, run-length, and texture energy metrics. These findings are consistent with the abrupt tissue disorganization, cellular swelling, and inflammation that characterize AKI, leading to greater local pixel variability and microstructural disruption [21]. AKI showed elevated importance in features such as GLDS_5 and WP_13, indicating more subtle microtextural changes likely related to edema or tubular injury rather than chronic remodeling. Healthy controls, on the other hand, demonstrated relatively lower and more uniform feature contributions, particularly in wavelet and GLSZM domains, reflecting more homogeneous parenchymal texture.

Echogenicity measures the overall brightness of the ultrasound image, which can be influenced by factors such as fibrosis, inflammation, and cellular debris [16]. The overlapping mechanisms underlying this increase may contribute to the inability to distinguish between the two conditions using this feature alone. Interestingly, echointensity significantly differed between the controls and CKD and AKI subjects but did not show significant differences between CKD and AKI. This suggests that echogenicity, while effective in distinguishing diseased kidneys from healthy ones, lacks the specificity needed to differentiate between CKD and AKI. This highlights the need for additional texture-based features.

In this study, we identified the top 10 radiomic features most responsible for variance across diagnostic groups using principal component analysis (PCA). These features, drawn from diverse texture families, including GLCM, GLSZM, FDTA, and wavelet packet transforms, effectively captured subtle patterns of tissue heterogeneity and microstructural organization that are often imperceptible on conventional grayscale ultrasound.

Using these selected features, we developed machine learning models that achieved notably high classification performance. Among them, XGBoost yielded the highest macro-averaged F1 score (0.90), with SVM and Random Forest also demonstrating robust performance. These findings highlight the diagnostic utility of radiomic signatures in stratifying kidney pathologies, especially in scenarios where imaging appearances may overlap and challenge traditional interpretation.

The application of UMAP for dimensionality reduction further reinforced the discriminative power of the selected features. The resulting two-dimensional projection revealed well-separated clusters corresponding to Acute Kidney Injury (AKI), Chronic Kidney Disease (CKD), and healthy controls, providing visual evidence of distinct radiomic phenotypes among the groups. This visual distinction confirms the capability of the PCA-selected features to preserve diagnostic group structure in a lower-dimensional space. The observed clustering supports the hypothesis that underlying renal pathology in AKI and CKD is reflected in distinct radiomic texture patterns, which can be quantitatively captured and visualized using machine learning techniques. These findings highlight the potential of radiomics-based dimensionality reduction and visualization tools in supporting automated diagnostic classification.

From a clinical standpoint, the ability to differentiate AKI from CKD using non-invasive, ultrasound-based radiomic features offers promising implications for early and accurate diagnosis. This is particularly valuable in pediatric populations or resource-constrained settings, where access to advanced imaging or invasive diagnostics may be limited. Moreover, the automated and reproducible nature of the radiomic workflow supports potential integration into clinical practice, especially as AI-driven ultrasound tools become increasingly accessible.

While this study demonstrates the utility of US radiomic features in differentiating CKD and AKI, several limitations warrant consideration. As a preliminary investigation intended to assess the effectiveness of radiomic techniques, our study included a relatively small sample size. This limitation, combined with the difference in age between subjects with kidney disease and controls, may have impacted our findings.

Furthermore, this study utilized retrospectively acquired clinical ultrasound images collected from different ultrasound machines and transducers. Consequently, variability in texture features across these devices might be present. Although such variations may exist, we believe that their overall impact on our results is minimal, preserving the robustness of our findings. Our ultrasound images were acquired using transducers and imaging settings standardly employed for renal assessments, ensuring clinical relevance.

In future work, we plan to prospectively collect data under more controlled conditions using a standardized ultrasound machine and transducer. Furthermore, we aim to implement additional image normalization techniques to enhance consistency in feature extraction, ultimately strengthening the radiomics model for clinical applications. Second, manual ROI selection may introduce variability, and future studies should explore automated segmentation techniques to enhance reproducibility. In this study, we employed PCA to identify the top 10 radiomic features for subsequent ML modeling. Typically, a ratio of approximately one feature per every 5–10 cases is recommended for robust modeling; thus, our selection of 10 features for 60 cases (approximately one feature per 6 cases) is within an acceptable but borderline range. While our models demonstrated strong performance, there remains a possibility of overfitting due to the limited sample size relative to the number of features used. Future validation studies with larger patient cohorts and external datasets are, therefore, recommended to confirm the generalizability of these features and ensure the robustness of the predictive models.

## 5. Conclusions

Our findings in this pilot study showed that ultrasound radiomics can differentiate between healthy controls’ kidney images, in addition to images taken from diseased patients, particularly acute kidney injury and chronic kidney disease. Texture features, combined with conventional echogenicity measures, can provide a powerful, innovative, and complementary characterization of the kidney and improve clinical decision-making. Further research is needed to refine these measurements and design clinically relevant diagnostic and prognostic biomarkers.

## Figures and Tables

**Table 1:** Selection criteria for the group cohorts.

	AKI	CKD	Healthy Controls
Criteria	KDIGO stage 3 with oligo-anuria Not on dialysis	CKiD study participants of cohorts I, II, and III [5]. Mild-moderate CKD Glomerular and non-glomerular disease 6 months to 17 years of age	No known history of kidney disease, urinary tract malformations, or febrile urinary tract infection (pyelonephritis) Body Mass Index between the 5th and 85th percentile Negative urine/serum pregnancy test before imaging
	Imaging indication: Oliguric/anuric AKI with elevated serum creatinine levels, meeting indications for dialysis for fluid overload and/or electrolyte management	Imaging indication: first ultrasound performed in participants who consented to have images analyzed	Imaging indication: first ultrasound performed in participants who consented to have images analyzed

**Table 2:** Participants’ demographic characteristics by groups.

Characteristics ^a^	AKI	CKD	Healthy Controls
Total, n (kidney units)	8 (16)	14 (28)	9 (18)
Female: n (%)	6 (75)	6 (42.9)	6 (66.7)
Median age in years (IQR)	3.5 (IQR: 0–11.5)	3.5 (IQR: 0–6.8)	15.5 (IQR: 12.8–21)
Mean serum creatinine ± SD	6.13 ± 6.13	0.76 ± 0.33	0.67 ± 0.22
Mean U25 eGFR mL/ min/1.73m^2^ ± SD	9.10 ± 8.48	61.98 ± 22.46	105.85 ± 25.21

^a^ Mean values are reported for variables with normal distributions, while median values are used for variables with skewed distributions.

**Table 3:** Top 10 radiomic features identified by Principal Component Analysis (PCA).

Feature Category	Feature Code	Description
Gray-Level Co-occurrence Matrix [GLCM]	GLCM_4 GLCM_5 GLCM_6	Second-order texture features quantifying spatial relationships between pixels
Gray-Level Difference Statistics [GLDS]	GLDS_3	Local texture features based on absolute gray-level differences
Fractal Dimension Texture Analysis [FDTA]	FDTA_1 FDTA_3	Measures of complexity and self-similarity based on fractal geometry
Gray-Level Size Zone Matrix (GLSZM)	GLSZM_2 GLSZM_3 GLSZM_9	Measures size and intensity homogeneity of connected pixel zones
Wavelet Packet [WP]	WP_14	Multiscale texture features extracted from wavelet packet decomposition

**Table 4:** Performance of Supervised Classification Models Using Top 10 PCA-Selected Radiomic Features.

Model (5-Fold CV)	Accuracy	Macro F1 Score
XGBoost	0.90	0.90
SVM (RBF Kernel)	0.90	0.88
Random Forest	0.88	0.89
Logistic Regression	0.88	0.88

## Data Availability

The datasets generated during and/or analyzed during the current study are available from the corresponding author upon reasonable request.

## Supplementary Materials

The following supporting information can be downloaded at: https://www.mdpi.com/article/10.3390/diagnostics15162112/s1, Table S1: This table summarizes the extracted ultrasound radiomic features categorized bytheir computational families. Each row lists the feature category, feature abbreviation (used for labelingand reference), and the corresponding feature name.
