# Supplementary material for: Quantifying “Medical Renal Disease”: A Pediatric Pilot Study Using Ultrasound Radiomics for Differentiating Acute Kidney Injury and Chronic Kidney Disease
Source: Diagnostics (Basel). 2025 Aug 21;15(16):2112. doi: 10.3390/diagnostics15162112 (PMC12385666; doi:10.3390/diagnostics15162112)
Supplement: Supplementary file 1 [file diagnostics-15-02112-s001.zip › diagnostics-3669734-supplementary.pdf]

Supplement Table S.1. This table summarizes the extracted ultrasound radiomic features categorized by their computational families. Each row lists the feature category, feature abbreviation (used for labeling and reference), and the corresponding feature name.

Asterisks (*) indicate features that were found to be statistically significant ($p < 0.05$) in differentiating at least one diagnostic group (AKI, CKD, or Control) based on post hoc pairwise comparisons following liner mixed effect analysis.

**Feature Category**	**Description**	**Feature abbreviation/no.**	**Feature Name**
First-Order Statistics [FOS]	Intensity-based descriptors capturing global distribution of voxel values	**FOS1***	Mean
		**FOS2***	Variance
		**FOS3***	Skewness
		**FOS4***	Kurtosis
		FOS5	Median
		FOS6	Energy
		FOS7	Entropy
		FOS8	RMS
		FOS9	Minimum
		**FOS10***	Maximum
		**FOS11***	MeanAbsDev
		**FOS12***	Range
		**FOS13***	Percentile_10
Gray-Level Co-occurrence Matrix [GLCM]	Second-order texture features quantifying spatial relationships between pixels	**GLCM1***	GLCM_Contrast
		**GLCM2***	GLCM_Dissimilarity
		GLCM3	GLCM_Homogeneity
		GLCM4	GLCM_AngularSecondMoment
		**GLCM5***	GLCM_Energy
		**GLCM6***	GLCM_Entropy
		GLCM7	GLCM_Correlation
		GLCM8	GLCM_SumAverage
		GLCM9	GLCM_SumVariance
		GLCM10	GLCM_SumEntropy
		GLCM11	GLCM_DifferenceVariance
		GLCM12	GLCM_DifferenceEntropy
		GLCM13	GLCM_InfoMeasureCorr1
		GLCM14	GLCM_InfoMeasureCorr2
		GLCM15	GLCM_MaxCorrCoef
		GLCM16	GLCM_ClusterTendency
		GLCM17	GLCM_ClusterShade
		**GLCM18***	GLCM_ClusterProminence
		GLCM19	GLCM_Autocorrelation
		**GLCM20***	GLCM_MCC

		**GLCM21*** **GLCM22***	GLCM_IDM GLCM_Shade
Gray-Level Difference Statistics [GLDS]	Local texture features based on absolute gray-level differences	**GLDS1*** GLDS2 **GLDS3*** GLDS4 **GLDS5*** GLDS6	GLDS_AngularSecondMoment GLDS_Entropy GLDS_Mean, GLDS_Dissimilarity GLDS_Contrast GLDS_Homogeneity
Neighborhood Gray-Tone Difference Matrix [NGTDM]	Texture metrics comparing gray levels to neighborhood averages	NGTDM1 NGTDM2 **NGTDM3*** **NGTDM4*** NGTDM5	NGTDM_Coarseness, NGTDM_Contrast NGTDM_Busyness NGTDM_Complexity NGTDM_Strength
Statistical Feature Matrix [SFM]	Spatial statistics derived from co-occurrence patterns	SFM1 SFM2 SFM3 SFM4	SFM_SF1 SFM_SF2 SFM_SF3 SFM_SF4
Law's Texture Energy [LTE]	Local texture patterns obtained via convolution with predefined kernels	**LTE1*** **LTE2 *** **LTE3 *** LTE4 LTE5 LTE6 LTE7 LTE8 **LTE9***	LTE_L5E5: Level – Edge LTE_L5S5: Level – Spot LTE_E5S5: Edge – Spot LTE_R5R5: Ripple – Ripple LTE_L5R5: Level – Ripple LTE_E5R5: Edge – Ripple LTE_S5R5: Spot – Ripple LTE_L5L5: Level – Level LTE_E5E5: Edge – Edge
Fractal Dimension Texture Analysis [FDTA]	Measures of complexity and self-similarity based on fractal geometry	**FDTA 1*** FDTA2 **FDTA 3*** FDTA4 FDTA 5 FDTA6	FDTA_Dim_mean FDTA_Dim_std FDTA_Dim_min FDTA_Dim_max FDTA_Dim_median FDTA_Dim_range
Gray-Level Run Length Matrix [GLRLM]	Quantifies consecutive runs of pixels with identical gray levels	**GLRLM1*** **GLRLM2*** GLRLM3	GLRLM_SRE (Short Run Emphasis, GLRLM_LRE (Long Run Emphasis GLRLM_GLN (Gray-Level Nonuniformity),

		GLRLM4	GLRLM_RLN (Run Length Nonuniformity),
		**GLRLM5***	GLRLM_LGRE (Low Gray-Level Run Emphasis),
		**GLRLM6***	HGRE (High Gray-Level Run Emphasis),
		**GLRLM7***	GLRLM_SRLGE (Short Run Low Gray-Level Emphasis),
		**GLRLM8***	GLRLM_SRHGE (Short Run High Gray-Level Emphasis),
		**GLRLM9***	GLRLM_LRLGE (Long Run High Gray-Level Emphasis),
		GLRLM10	GLRLM_LRHGE (Long Run High Gray-Level Emphasis
		**GLRLM11***	GLRLM_RP (Run Emphasis)
Fourier Power Spectrum [FPS]	Frequency-domain features capturing texture periodicity	**FPS1***	FPS_Slope,
		**FPS2***	FPS_Intercept
		**FPS3***	FPS_Energy
		**FPS4***	FPS_FractalDim
Shape Features	Morphological descriptors of the segmented region of interest	**Shape1***	Shape_Area
		**Shape2***	Shape_Perimeter
		**Shape3***	Shape_Compactness
		Shape4	Shape_Circularity
		**Shape5***	Shape_Convexity
Gray-Level Size Zone Matrix (GLSZM)	Measures size and intensity homogeneity of connected pixel zones	**GLSZM1***	GLSZM_SAE
		**GLSZM2***	GLSZM_LAE
		**GLSZM3***	GLSZM_LGZE
		**GLSZM4***	GLSZM_HGZE
		**GLSZM5***	GLSZM_SALGLE
		**GLSZM6***	GLSZM_SAHGLE
		**GLSZM7***	GLSZM_LALGLE
		**GLSZM8***	GLSZM_LAHGLE
		**GLSZM9***	GLSZM_GLV
		GLSZM10	GLSZM_ZSV
		**GLSZM11***	GLSZM_ZP
Higher-Order Spectra (HOS)	Nonlinear spectral features including higher-order moment statistics	**HOS1***	HOS_Skewness,
		**HOS2***	HOS_Kurtosis,
		HOS3	HOS_Bispectrum1
		HOS4	HOS_Bispectrum2

Local Binary Pattern (LBP)	Encodes local texture by comparing each pixel with its neighbors	**LBP 1***<br>**LBP2***<br>LBP3<br>LBP4<br>LBP5<br>LBP6<br>**LBP7***<br>LBP8<br>LBP9	LBP_P8R1<br>LBP_P8R1_uniformity<br>LBP_P16R2<br>LBP_P16R2_uniformity<br>LBP_P24R3_uniformity<br>LBP_hist_entropy<br>LBP_P24R3<br>LBP_hist_energy,- missing<br>LBP_hist_mean
Wavelet Packet [WP]	Multi-scale texture features extracted from wavelet packet decomposition	WP1<br>WP2<br>WP3<br>WP4<br>WP5<br>WP6<br>WP7<br>WP8<br>WP9<br>**WP10***<br>WP11<br>**WP12***<br>WP13<br>**WP14***<br>WP15	WP_1: Global average intensity (smooth background information)<br>WP_2: Low-frequency vertical + high-frequency horizontal<br>WP_3: High-frequency vertical + low-frequency horizontal<br>WP_4: Diagonal edges in low-frequency context<br>WP_5: Mid-high vertical edges on smooth horizontal texture<br>WP_6: High-frequency horizontal features (textural detail)<br>WP_7: High-frequency vertical features (fine structure)<br>WP_8: High-frequency diagonal features (fine structure)<br>WP_9: High horizontal, low vertical (textural shifts)<br>WP_10: Edge enhancement in horizontal components<br>WP_11: Texture irregularities with vertical orientation<br>WP_12: High-frequency diagonal noise or fine texture<br>WP_13: Very fine edge components preserved after smoothing<br>WP_14: Micro horizontal patterns<br>WP_15: Micro vertical patterns
		**124**	
